# MRI Predicts Outcome After HIE Treated with Hypothermia

**DOI:** 10.15844/pedneurbriefs-29-2-2

**Published:** 2015-02

**Authors:** Maura E. Ryan

**Affiliations:** 1 Division of Neuroradiology, Department of Medical Imaging, Ann & Robert H. Lurie Children's Hospital of Chicago, Chicago, IL; 2 Department of Radiology, Northwestern University Feinberg School of Medicine, Chicago, IL

**Keywords:** Hypothermia, Corpus Callosum, Magnetic Resonance Imaging, Diffusion Tensor Imaging

## Abstract

Investigators from Children's National Medical Center and George Washington University School of Medicine, Washington, DC, studied the correlation between white matter tract changes and developmental outcomes in a series of infants with hypoxic ischemic encephalopathy (HIE) treated with whole body cooling.

Investigators from Children's National Medical Center and George Washington University School of Medicine, Washington, DC, studied the correlation between white matter tract changes and developmental outcomes in a series of infants with hypoxic ischemic encephalopathy (HIE) treated with whole body cooling. Using magnetic resonance imaging (MRI) with quantitative diffusion tensor imaging (DTI), fractional anisotropy, as well as mean, axial and radial diffusivity of the corpus callosum (CC), posterior limbs of the internal capsule (PLIC) and cortical spinal tracts (CST) were calculated in 50 infants at a median age of 8 days of life, and compared with neurodevelopmental outcomes using a Psychomotor Developmental Index (PDI) and Mental Developmental Index (MDI) derived from the Bayley Scales of Infant Development at 15 months (42 patients) and 21 months (35 patients) of age.

Controlling for gestational age, birth weight, socioeconomic status and gender, there was a statistically significant correlation (p<0.001) between decreased fractional anisotropy of the CST and lower PDI scores as well as decreased fractional anisotropy of the CC and lower MDI scores. Increased axial diffusivity of the CC and lower radial diffusivity of the CST also correlated with lower PDI and MDI scores (p=0.018 and p=0.03respectively). [1]

COMMENTARY. HIE is a common cause of mortality and long-term morbidity in neonates. Early assessment of the degree of neurologic injury is important for prognosis and rehabilitation planning, but can be challenging by conventional imaging methods. Evaluation by standard MRI is complicated by observer variability as well as significant differences in imaging findings depending on the timing of the exam [2, 3].

Diffusion Tensor Imaging (DTI) is an advanced magnetic resonance imaging technique that uses the restricted movement of water molecules perpendicular to the long axis of white matter tracts as a measure of axonal integrity and a decrease in fractional anisotropy in HIE patients suggests microstructural injury [4]. DTI may prove to be a useful tool in evaluating the severity of white matter injury in HIE patients, although the clinical applications of the data are limited by the small sample size and complexities of incomplete myelination in children.
